# Role of plasma levels of CA-125 in predicting outcome of primary PCI after acute myocardial infarction in male patients

**DOI:** 10.15171/jcvtr.2018.17

**Published:** 2018-05-21

**Authors:** Ahmad Separham, Mohsen Abbasnezhad, Golnesa Shahnazarli, Alireza Khoshbahar

**Affiliations:** ^1^Cardiovascular Research Center, Tabriz University of Medical Sciences, Tabriz, Iran; ^2^Department of Midwifery and Nursing, Midwifery Faculty, Tabriz University of Medical Sciences, Tabriz, Iran

**Keywords:** Acute Myocardial Infarction, CA-125, Outcome

## Abstract

***Introduction:*** Cancer antigen 125 (CA-125) is a tumor marker of ovarian cancer, which has shown to be increased in different cardiovascular diseases. Although the prognostic role of CA-125 in heart failure and coronary heart disease is well-established, there is little known about its role in acute myocardial infarction (AMI). In this study we aimed to evaluate the serum levels of CA-125 in patients with AMI and its prognostic role in evaluating the in-hospital outcome of AMI.

***Methods:*** We evaluated 120 male patients with AMI and 120 male normal subjects. CA-125 levels were measured upon the patient’s admission to hospital. The in-hospital major adverse cardiac events (MACE) and its predictors were also recorded for AMI patients.

***Results:*** CA-125 levels were significantly higher in AMI patients compared to normal subjects (7.99±6.83 vs. 5.70±4.62, P = 0.003). We found significant positive correlations between CA-125 levels with creatine kinase-MB (CKMB) (r=0.621, P < 0.001) and CTnI (r=0.491, P < 0.001). The in-hospital MACE was observed in 19 cases (15.8%). Patients with MACE had significantly higher value of CA-125, CKMB and CTnI and lower LVEF compared to patients without MACE. CKMB (OR=0.967, 95% CI [0.943-0.991], P = 0.007) and CA-125 levels (OR=0.821, 95% CI [0.688-0.979], P = 0.02) were independent predictors of MACE.

***Conclusion:*** Serum CA-125 levels are significantly higher in male patients with AMI compared to normal subjects and have a significant role in predicting in-hospital MACE after AMI. In patients with higher CA-125 more aggressive treatment and close observation should be performed in order to reduce the possible adverse outcomes.

## Introduction

Cancer antigen 125 (CA-125) is produced by endometrial, peritoneal, and ovarian cells, which is normally used as a tumor marker of ovarian cancer.^1^ Besides the role in monitoring ovarian cancer therapy, high CA-125 levels is reported in both ovarian and non-ovarian diseases, malignant and nonmalignant conditions.^2,3^ The exact biological role of CA-125 in unknown, but it seems to act through several pathways due to its complex structure.^4^

CA-125 is considered to be a diagnostic and prognostic marker in cardiovascular diseases. In recent years, various studies have reported prognostic role of increased CA-125 in different cardiac diseases.^4-7^ Increased levels of CA-125 are also shown in patients with acute coronary syndrome and systolic dysfunction or acute heart failure.^8^

Due to these findings, it is possible that CA-125 could be used as prognostic factor in myocardial infarction (MI). There are few previous studies reporting increased CA-125 levels in patients with MI.^7-9^ In this study, we measured CA-125 levels in patients with acute MI and its prognostic role in evaluating the outcome of MI.

## Materials and Methods

In this case control study, 120 eligible male patients admitted to Madani Heart Center, Tabriz, Iran between August 2014 and December 2014 with ST elevation acute MI (STEMI) who underwent primary percutaneous coronary intervention (PCI) were recruited. Control group were 120 male subjects who were evaluated for suspicion of heart disease with normal cardiac findings. Both groups were matched for baseline findings. As CA-125 is increased in genital disease in female patients including ovarian disease, to decrease the possible bias in Ca-125 levels, we only recruited male subjects. Exclusion criteria were history of congenital heart disease, congestive heart failure (CHF), vascular or valvular disease, cardiomyopathy, left ventricular systolic dysfunction with non-ischemic causes, atrial fibrillation, previous STEMI, regional-wall motion abnormalities in non–infarct-related regions, previous coronary artery bypass grafting (CABG), pleural or pericardial effusion, ascites, active infection requiring intravenous antibiotics, chronic renal or liver disease, malignancy, hematological disorders.

Study sample was calculated n = 109 for each group using G*Power 3.1.3.9 software considering the effect size of *d* ≥ 0.30 as statistically significant in a two-tailed test with α=0.05 and power of 0.95. In order to reach better results, we included 120 patients in each group.

Patients’ demographic, laboratory and clinical data were collected. All patients were classified by Killip classification^10^ at the baseline. Left ventricular ejection fraction (LVEF) was measured by transthoracic echocardiography.

All patients underwent PCI and the in-hospital outcome and major adverse cardiac events (MACE) including cardiac and non-cardiac death, MI, HF and need for revascularization were recorded.

Blood samples were collected at the admission. Creatine kinase-MB (CKMB) was measured in three different periods and CTnI was measured twice and the peak CKMB and CTnI was included. CA-125 was measured with chemiluminescent enzyme immunoassay methods by using an Acculite CLIA, commercial kit (Monobind, USA). CA-125 normal level was 35 UI/mL.

### Data analysis

All data were analyzed using SPSS statistics version 17.0. Numerical variables were expressed as mean ± standard deviation (SD), whereas categorical variables were expressed as percentage. The chi-square, Fisher exact test, Student’s *t* test and Mann-Whitney U test were used to compare findings between groups. Correlations were analyzed using the Pearson correlation. Logistic regression analysis was used to define predictors of in-hospital MACE. A *P* value <0.05 was considered statistically significant.

## Results

Table 1 demonstrates demographic findings and CA-125 levels of patients and controls. Both groups were comparable regarding age and cardiovascular risk factors, while acute myocardial infarction (AMI) patients had significantly higher CA-125 levels.

**Table 1:** Demographic findings and CA-125 levels of patients and controls

	**AMI Group** **(n=120)**	**Controls** **(n=120)**	***P*** value*
Age (y)	56.84±8.73	56.55±8.78	0.79
Hypertension	60 (50%)	63 (52.5%)	0.69
Diabetes mellitus	55 (45.8%)	48 (40%)	0.36
Hyperlipidemia	36 (30%)	42 (35%)	0.40
Smoking	37 (30.8%)	33 (27.5%)	0.57
Familial history of coronary heart disease	19 (15.8%)	12 (10%)	0.17
CA-125 (U/mL)	7.99±6.83	5.70±4.62	0.001

* *P* is two-sided significant. Independent *t* test or Mann-Whitney U test, Chi-square test and Fisher’s exact are used.

Most patients were in Killips class I (110, 91.7%) and few were in Killips class II and III (6 [5%] and 4 [3.3%], respectively). Patients had mean LVEF of 42.29±9.52%. We found significant correlations between CA-125 levels with CKMB (r=0.621, *P *< 0.001) and CTnI (r=0.491, *P *< 0.001), while the correlations with other variables were not significant in patients with AMI.

The in-hospital MACE was observed in 19 cases (15.8%) including Re-MI in 6 cases, revascularization in 11 cases, CABG in 2 cases, cardiac death in 2 and overall death in 4 cases. Few patients had 2-3 complications during the hospital stay. There were no cases of major bleeding or stroke. There were also no cases of new onset atrial fibrillation or HF after MI during the hospital stay. Demographic and laboratory findings among patients with and without MACE were evaluated and there was significantly higher levels of CA-125, CKMB and CTnI and lower LVEF in patients with MACE compared to those without MACE (Table 2).

**Table 2:** Demographic findings and laboratory findings of patients with and without MACE

	MACE (n=19)	No MACE (n=101)	P value*
Age (y)	60.11±9.43	56.23±8.50	0.07
Hypertension	11 (57.9%)	49 (48.5%)	0.45
Diabetes mellitus	10 (52.6%)	45 (44.6%)	0.51
Hyperlipidemia	7 (36.8%)	29 (28.7%)	0.47
Smoking	3 (15.8%)	34 (33.7%)	0.12
Familial history of CHD	2 (10.5%)	17 (16.8%)	0.49
LVEF	38.15±10.16	43.06±9.24	0.03
CA-125 (U/mL)	18.92±9.17	6.01±3.67	<0.001
CKMB	297.47±93.58	166.85±62.42	<0.001
CTNI	14.55±9.34	7.27±4.53	<0.001

Abbreviations: MACE, major adverse cardiac events; CHD, coronary heart disease; LVEF, left ventricle ejection fraction; CKMB, creatine kinase-MB; CTnI, cardiac troponin I. * *P* is two-sided significant. Student’s t test and Mann-Whitney U test are used.

Significant findings were used in logistic regression analysis to find predictors of MACE (Table 3) and observed that only CKMB (OR=0.967, 95% CI [0.943-0.991], *P *= 0.007] and CA-125 levels (OR=0.821, 95% CI [0.761-1.059], *P *= 0.02) were independent predictors of MACE. In fact, lower levels of CKMB and CA-125 had protective effect in occurrence of MACE.

**Table 3:** Logistic regression analysis to define predictors of MACE

	**OR**	**CI**		***P*** Value*
		**Lower limit**	**Upper limit**	
LVEF	1.093	0.994	1.203	0.06
CKMB	0.967	0.943	0.991	0.007
CTnI	0.898	0.688	0.979	0.20
CA-125	0.821	0.761	1.059	0.02

Abbreviations: OR, Odds ratio; LVEF: left ventricle ejection fraction; CKMB: creatine kinase-MB; CTnI: cardiac troponin I. * *P* is two-sided significant.

## Discussion

The increased CA-125 levels have been reported in different cardiac pathologies including CHF, coronary heart disease (CHD), AF and valve disease such as aortic stenosis and mitral stenosis.^5-7,11,12^ Although there are few reports, the increased CA-125 levels is also documented in AMI.^7-9^

In this prospective study, we observed that AMI patients compared to normal subjects had significantly higher levels of CA-125 levels. The outcome of AMI was poor in older patients and in those with lower LVEF. Higher levels of CA-125, CKMB and CTnI and lower LVEF were found in patients with MACE than without MACE, of which CKMB and CA-125 were independent predictors of MACE. Husser et al^13^ also reported that CA-125 levels before and after aortic valve implantation could independently predict death and MACE.

There are some possible mechanisms in which CA-125 could be increased in cardiac disease. The role of hemodynamic abnormalities and inflammatory cytokines on the CA-125 levels have been recommended in malignant, non-malignant and chronic diseases like CHF.^3-7^ These may also have a role in the development of atherosclerosis and its complications.^14^ Mechanical stress and inflammation could induce CA-125 synthesis from the mesothelial cells of the peritoneum, pleura, and pericardium.^11,15^ Considering these changes, CA-125 could act by inflammatory repose in CHD development and also AMI occurrence.

Although myocardial production of CA-125 have not been documented, the possibility could not be excluded. Rong and colleagues^7^ have suggested that ischemic injury of myo­cardium causing possible ventricular remodeling and heart enlargement would have a role in the secre­tion of CA125 in CHD patients. However, these findings need more investigations.

While the exact mechanism of CA-125 level increase in cardiac disease is not well understood, there are some properties that make CA-125 a promising prognostic tool: it is widely available and less expensive that other biomarkers. It is stable with long half life (more than a week) which is correlated with clinical status and prognosis.^16^ In fact, CA-125 has shown considerable correlation to functional and clinical status of the patient and may demonstrate those at risk of adverse outcomes.^17^

## Limitations

There were several limitations. This was a single center study that would limit to extend it to the general population. Also, the sample of patients and number of outcome, especially mortality rate were small. We did not recheck the CA-125 levels after the acute phase of the AMI to compare the possible changes in its levels and define its exact role in STEMI which could be another limitation of our study.

As in this study we excluded women from the study to reduce the possible effects of gender and women related disease on CA-125 levels, it could be concluded that there is higher CA-125 levels in male patients with AMI and have significant role in predicting in-hospital MACE after AMI. In patients with higher CA-125 more aggressive treatment and close observation should be performed in order to reduce the possible adverse outcomes.

## Ethical approval

The Ethics committee of Tabriz University of Medical Sciences approved the study and in-formed consent were obtained from all participants.

## Competing interests

None.

## Funding

This article was supported by Cardiovascular Research Center, Tabriz University of Medical Sciences, Tabriz, Iran. Authors have no other funding support to disclose
